# Bioinformatics approaches for cross-species liver cancer analysis based on microarray gene expression profiling

**DOI:** 10.1186/1471-2105-6-S2-S6

**Published:** 2005-07-15

**Authors:** H Fang, W Tong, R Perkins, L Shi, H Hong, X Cao, Q Xie, SH Yim, JM Ward, HC Pitot, YP Dragan

**Affiliations:** 1 Division of Bioinformatics, Z-Tech Corporation, 3900 NCTR Road, Jefferson, AR 72079; 2 Division of Systems Toxicology, National Center for Toxicological Research (NCTR), FDA, 3900 NCTR Road, Jefferson, AR 72079; 3 Laboratory of Metabolism, Center for Cancer Research, National Cancer Institute, NIH, Bethesda, Maryland 20892; 4 Verterinary and Tumor Pathology Section, Center for Cancer Research, National Cancer Institute, Frederick, Maryland 21702; 5 McArdle Laboratory for Cancer Research, University of Wisconsin, Madison, WI 53706

## Abstract

**Background:**

The completion of the sequencing of human, mouse and rat genomes and knowledge of cross-species gene homologies enables studies of differential gene expression in animal models. These types of studies have the potential to greatly enhance our understanding of diseases such as liver cancer in humans. Genes co-expressed across multiple species are most likely to have conserved functions. We have used various bioinformatics approaches to examine microarray expression profiles from liver neoplasms that arise in albumin-SV40 transgenic rats to elucidate genes, chromosome aberrations and pathways that might be associated with human liver cancer.

**Results:**

In this study, we first identified 2223 differentially expressed genes by comparing gene expression profiles for two control, two adenoma and two carcinoma samples using an F-test. These genes were subsequently mapped to the rat chromosomes using a novel visualization tool, the Chromosome Plot. Using the same plot, we further mapped the significant genes to orthologous chromosomal locations in human and mouse. Many genes expressed in rat 1q that are amplified in rat liver cancer map to the human chromosomes 10, 11 and 19 and to the mouse chromosomes 7, 17 and 19, which have been implicated in studies of human and mouse liver cancer. Using Comparative Genomics Microarray Analysis (CGMA), we identified regions of potential aberrations in human. Lastly, a pathway analysis was conducted to predict altered human pathways based on statistical analysis and extrapolation from the rat data. All of the identified pathways have been known to be important in the etiology of human liver cancer, including cell cycle control, cell growth and differentiation, apoptosis, transcriptional regulation, and protein metabolism.

**Conclusion:**

The study demonstrates that the hepatic gene expression profiles from the albumin-SV40 transgenic rat model revealed genes, pathways and chromosome alterations consistent with experimental and clinical research in human liver cancer. The bioinformatics tools presented in this paper are essential for cross species extrapolation and mapping of microarray data, its analysis and interpretation.

## Background

For decades, classical toxicology has used risk assessments based on animal studies for regulatory decisions. The underlying assumption is that important biological functions are often conserved across species. In continuation of this paradigm, the effort in toxicogenomics is placed on studying rodents and other surrogates using advanced genomics technologies, such as DNA microarrays. Microarray studies enable simultaneous measurement of the expression of large numbers of genes. Given the completion of the DNA sequence of the human, mouse and rat genomes [1-3], genes identified in microarray studies can be readily compared across-species with respect to the gene orthologs [4,5]. This assumes that genes co-expressed across multiple species are likely to have conserved functions [6-8]. Thus, microarray analysis offers the possibility of furthering our understanding of cross-species commonalities and differences that could lead to more effective use of animal models to understand the cause and progression of diseases in human at the mechanistic level.

Hepatocellular carcinoma (HCC) is a leading cause of death worldwide and, like most cancers, is a genetic disease caused by the accumulation of genetic and epigenetic cell alterations. The progression of hepatic neoplasia is characterized by increasing genetic instability, including duplication and deletion of parts of chromosomes and an increasing proliferative growth advantage of the affected cells. Molecular cytogenetic techniques, such as Comparative genomic hybridization (CGH) and Spectral karyotyping (SKY) [9-11], have allowed evaluation of chromosomal aberrations in HCC. More recently, Crawley [12] has demonstrated the ability of comparative genomic microarray analysis (CGMA) to elucidate alteration of specific genes together with the genetic changes at the chromosome level based on microarray data. Thus, microarray analysis provides an unprecedented opportunity to further the understanding of the etiology and progression of liver cancer.

Bioinformatics methods and tools are essential to analyze and interpret data from microarrays. The critical and urgent task is to associate altered patterns of gene expression with disease. Interpreting microarray data in the context of signaling and regulatory pathways is a particularly effective bioinformatics approach to transform data into biological meaning and to generate hypotheses for further research. Using pathways, disease mechanisms can be interpreted as disturbances of the intricate interconnections among genes, molecules and cells. Most reported pathway analysis of microarray data has examined the role of differentially expressed genes in pathways selected with *a priori *knowledge. Alternatively, significant pathways can be identified based on statistical analysis, potentially leading to new discoveries and a more complete interpretation of microarray data in the context of biological processes at the mechanistic level.

The primary mechanism for the analysis of HCC is by the administration of carcinogenic agents. A number of model systems have been developed to understand the pathogenesis of primary liver cancer [13-15]. Additionally, the development of transgenic models permit the analysis of the genetic basis for the induction and progression of HCC [16-19]. The albumin-Simian virus 40 (SV40) T antigen transgenic rat contains the mouse albumin-promoter/enhancer linked to the coding region of the SV40 large T antigen (SV 40 tag). SV40 T antigen inactivates both p53 and Rb, resulting in spontaneous development of hepatic neoplasms (adenoma and carcinoma) within 6–9 months. Thus, the Albumin-SV40 T antigen transgenic rat can be used to examine liver cancer development and maintenance [20-22].

In this manuscript, we describe a bioinformatics process where microarray data from the SV40 transgenic rat was examined for application to the study of HCC in human. We first used a novel visualization tool to investigate liver cancer by mapping chromosomal location of differentially expressed genes from the rat model to the chromosomal regions of human orthologs. Then, CGMA analysis was used to relate gene expression bias patterns to cytogenetic aberration profiles on human. Lastly, a statistical approach was used to identify several pathways involved in human HCC based on the rat microarray. The pathway analysis reveals that the expected involvement in apoptosis, cell cycle, growth and differentiation, genetic stability and methionine metabolism are important for cancer development, maintenance and progression. The results indicate that the gene expression profiles of the transgenic rat model may be useful in the study of human liver cancer.

## Methods

### Microarray experiment and results

The details of the microarray experimental procedure is reported elsewhere[21]. Briefly, RNA samples were isolated from the rat liver tissues of six samples, two controls, two adenomas and two carcinomas. The laser capture microdissected samples were amplified prior to microarray hybridization. An NCI cDNA array (IncyteGem2) was used that contains 10238 probes representing 9984 unique genes. Gene expression profiles were produced for all six samples with dye flip, which resulted in a total of 12 arrays.

The log2 ratio-based mean global normalization was first applied and the normalized ratios of the swapped dye labels were then averaged. A total of 9150 genes remained for further analysis after removing non hybridized genes due to low intensity. Significantly differentially expressed genes were determined using an F-test with P < 0 .05.

### Data analysis using ArrayTrack

**Figure 1:**
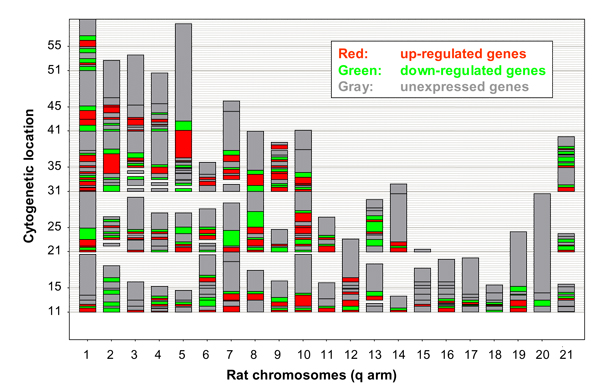
**Expressed genes in microarray mapped to the rat cytogenetic location and chromosome. **The genes were obtained from an ANOVA analysis among two controls, two adenomas and two carcinomas samples of the transgenic rat. The cytogenetic location of genes is on the y-axis for each of the 20 rat chromosomes that are displayed as separate bars along the x-axis. Red and green areas are the significant genes that are up or down regulated, respectively, and grey represents those genes not differentially expressed.

**Figure 2:**
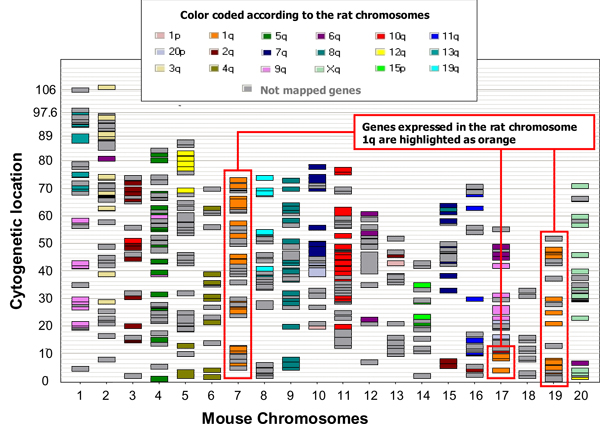
**Genes significantly differentially expressed in rat (shown in Figure 1) mapped to the orthologous genes on chromosome of mouse. **Different colors denote the corresponding rat chromosome number of the orthologous genes

**Figure 3:**
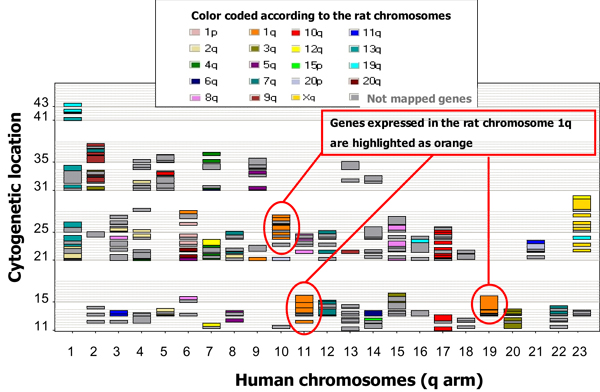
**Genes significantly differentially expressed in rat (shown in Figure 1) mapped to the orthologous genes on chromosomes of human. **Different colors denote the corresponding rat chromosome number of the orthologous genes.

Most analyses were conducted using in-house software, ArrayTrack . ArrayTrack is bioinformatics software, where data management, analysis and interpretation are fully integrated [37]. ArrayTrack consists of three components: (1) MicroarrayDB for storing microarray data; (2) LIBRARY for data interpretation that contains many types of functional information about genes, proteins and pathways; and (3) TOOL that provides functionality for microarray data analysis. LIBRARY contains many sub-libraries and data in these sub-libraries is extracted from different biological databases in public domain (e.g., NCBI bioinformatics resources)[38]. In this project, information for orthology analysis, chromosome-based analysis and pathway analysis was retrieved from LIBRARY. More specifically,

• Gene Orthology Analysis – The human and mouse orthologs to rat were obtained from the Orthologene Library in ArrayTrack. The content of the Orthologene Library are mainly derived from the NCBI HomoloGene database [4,5]. ArrayTrack allows rapid matching of a large number of genes across human, mouse and rat for the gene orthology analysis.

• Chromosome-based analysis – The cytogenetic locations of genes were exported directly from the Gene Library of ArrayTrack. A novel visualization tool, the Chromosome Plot, was developed to study the effect of a gene expression pattern on liver cancer through identifying the altered cytogenetic regions of each chromosome. Figure 1 shows a bar chart depiction with the y-axis giving cytogenetic location along each rat chromosome represented by 20 vertical bars extending along the x-axis. This kind of plot has two uses. It depicts rat genes in their cytogenetic locations on each chromosome using color coding expression information as red for up-regulation, green for down and grey for unaffected genes (e.g., Figure 1). Thus, the plot provides for a specific species a compact visual display of cytogenetic blocks and/or chromosomes altered. Alternatively, the genes can also be mapped to the chromosomal location of another species, and color coded according to the chromosome of the experiment species. (e.g., Figures 2 for rat mapping to mouse and Figure 3 for rat mapping to human).

• Pathway analysis – The pathway data was obtained from the Pathway Library in ArrayTrack. The Pathway Library contains pathways from both the Kyoto Encyclopedia of Genes and Genomes (KEGG)[35] and PathArt (Jubilant Biosys Ltd, Columbia, MD 21045) that can be searched separately or in combination in ArrayTrack. The Fisher Exact Test [39] was used to estimate the statistical significance of pathway *i*:



Where *N *is total number of genes on the chip (i.e., 9150), *m *is the number of differential expressed genes identified using the F-test (i.e., 2223), *n*_*i *_is the number of genes out of *N *that belong to pathway *i *while *m*_*i *_is the number of genes out of *M *differential expressed genes belong to pathway *i*. The two-sided Fisher's Exact Test p-value less than 0.05, suggest that the probability of significant genes in this pathway is not expected by chance alone.

### Comparative Genomic Microarray Analysis (CGMA)

CGMA identifies cytogenetic regions containing unidirectional gene expression biases. The biased regions possibly indicate chromosomal gains and losses [12]. Of the total 9150 genes, GenBank accession numbers (Refseq in NCBI) for human orthologs to rodent genes were obtained for 2925 genes out of 3414 genes with Homologene ID using the Orthologene Library and Gene Library in ArrayTrack; ESTs and genes that may be unique to rodent were excluded. A two-tailed z-statistic was then computed to test whether chromosomal regions exhibited gene-expression biases [12]. CGMA was done for each of two adenoma samples and each of the two carcinoma samples using an on line version of software at . Output human CGMA results contained 2728 genes from an input of 2925 Refseq genes. A Z-statistics of 1.96 corresponds to 95% confidence (that the expression bias in the chromosome was not due to chance) and 2.58 corresponds to 99% confidence.

## Results

A total of 2223 differentially expressed genes was identified across three groups of samples (i.e., normal, adenoma and carcinoma) based on an F-test. The differentially expressed genes were first mapped to the rat chromosome. As depicted in Figure 1, the differentially expressed genes primarily occurred in several chromosomes, indicating that these chromosomes were altered in rats with neoplasm compared to normal rats. Specifically, a large number of up-regulated genes mapping in the rat chromosome 1q is consistent with previous findings of high amplification in rat liver cancer [22].

To investigate the cross-species extrapolation based on the results from the transgenic rat, the differentially expressed genes were first mapped to the orthologous chromosomal location of the mouse chromosomes. As depicted in Figure 2, the majority of the differentially expressed genes from the rat 1q that are known to be important for the rat liver cancer development appear mainly on the mouse chromosomes 7, 17, and 19 (displayed as the orange band in Figure 2). A comparison of rat to human shows that the differentially expressed genes from the rat 1q appear primarily on human chromosomes 10, 11 and 19 (Figure 3). The results suggest that the mouse chromosomes (7, 17 and 19) and human chromosomes (10, 11 and 19) might be important in liver cancer for these species. The findings are supported by a number of reports [12,23,24].

Table 1 lists the cytogenetic location of the differentially expressed genes from the rat 1q and location of the orthologous gene in human and mouse. There are seven groups of significantly expressed genes (called gene blocks); genes in each group are consecutive to each other and across species. The genes in the same blocks could be coordinately expressed to perform similar transcriptional programs or physiological processes across species in liver cancer development and maintenance. For example, the human gene blocks 10q24-26, 11p15.5, 11q13-15 and 19q13.2 have corresponding blocks on rat 1q, and corresponding blocks on mouse chromosomes 7 and 19. These blocks are associated with several cancer-related processes and functions, including apoptosis, M phase, cell communication and nuclear division as seen in a statistical analysis based on Gene Ontology (results not shown).

To further confirm the validity of cross-species extrapolation, we investigated chromosomal aberration in human based on the differentially expressed genes from the rat model using CGMA. Table 2 summarizes the Z statistics for each cancer sample from the CGMA analysis. Chromosomes exhibiting unidirectional bias with at least 95% confidence have the table cells with positive value denoting up-regulation or cells with negative vaule denoting down-regulation. Of 46 chromosomal regions (23 p and 23 q arms), 15 exhibit unidirectional bias in gene expression. Of the 15 affected chromosomal regions, 14 show up-regulation and most of these are associated with adenoma. The CGMA results were further compared with Karyotype results in the Cancer Genome Anatomy Project (CGAP) in the Mitelman Database [25]. Of 15 affected chromosomal regions identified from of rat gene expression data, 10 regions are also reported in CGAP. This is shown in the last column of Table 2 that lists both the number of citations and number of patients in CGAP.

We also investigated which pathways in human were significantly affected based on the differentially expressed genes identified in the transgenic rat model. Pathway analysis is a particular effective way to examine how the findings in the rat model relate to human in the context of biological functions. Table 3 summarizes the results of the pathways analysis. Fifteen pathways were significantly altered in a Fisher's Exact Test with p < 0.05. They predominately involve cell cycle, cell growth and differentiation. Most identified pathways are confirmed by a large literature to be associated with many cancers types [26,27]. Examples are 1) the p53 pathway involved in response to DNA damage, 2) the Rb pathway involves in the control of cell cycle, and 3) the transforming growth factor-beta (TGF-beta) pathway involved in growth inhibition. In addition, altered methionine metabolism pathway and regulation of P27 during cell cycle progression are known to be critical for cancer progression [28].

## Discussion

This study investigates the implications of using microarray results from the albumin-SV40 transgenic rat for the study of human liver cancer. We demonstrated the importance of bioinformatics to interpret microarray data for the cross-species comparisons. Specifically, two in-house bioinformatics tools are of importance for the analysis, the Chromosome Plot and ArrayTrack. The Chromosome Plot not only provides a visual presentation of the gene expression pattern at the level of gene order across chromosomes (e.g., Figure 1), but also can be used to map chromosome and cytogenetic location of differentially expressed genes from one species to another (e.g., Figures 2 and 3). ArrayTrack software that integrates data from public repositories was used to identify the cross-species orthologous genes, their chromosomal locations and, most importantly, the pathways that may be related to liver cancer. In addition, CGMA analysis was performed to investigate the variability of the multiple chromosome aberration patterns based on gene expression data, which is compared with the results presented in CGAP.

### Implications of orthologs and chromosome-based analysis

The recently completed sequencing of the rat genome provides a basis for future research to elucidate how differences and commonalities affect the ability of rat models to predict human disease. The rat genome project reported that almost all human genes known to be associated with disease have orthologous genes in the rat genome, and that human, mouse and rat genomes are approximately 90% orthologous [1]. We also analyzed orthologous genes between human, rat and mouse among the 9150 genes on the chip using ArrayTrack. The chip was found in Orthologene Library in ArrayTrack to contain 3414 human, 3365 mouse and 1950 rat genes, with the rest of genes being either EST tags or Riken genes (about 1500). The results showed that 92% of human genes are orthologous to either rat or mouse.

Although a large number of genes was identified to be differentially expressed from the rat model, some of these genes may result from the cancer rather than causally related. In addition, the function of a specific gene and its involvement in cancer might not be conserved across species. Thus, as important as structural and functional homology of specific genes is, the conservation of function of blocks of genes is likely to be more important in cross-species comparison. We found seven distinct blocks of significantly differentially expressed genes within different cytogenetic regions of the rat 1q with homologous chromosomal segments in human and mouse (Table 1). However, human, mouse and rat have very different chromosomal arrangements. The genes in these blocks appear consecutively in contiguous cytogenetic regions, irrespective of species and chromosomal location. This finding is not surprising considering the close evolutionary distance between the species where 278 orthologous segments are reported to be shared between human and rat, and 280 segments are reported to be shared between human and mouse [1].

It is proposed that these seven blocks of genes may be of significance for liver cancer development, maintenance and progression across human, rat, and mouse. For example, genes in the blocks may be coordinately expressed to share transcription programs or to respond to the genomic instability observed in liver cancer. Several genes in Table 1 show large fold changes, and are implicated in cancer development and maintenance. For example, Rps16, Rps19 and Rps3 code for ribosomal proteins, and their altered expression has been associated with liver and other tumors [29,30]. Insulin-like growth factor2 receptor (Igf2r) is mutated in many human HCC tumors and the gene's haploid insufficiency has been suggested as an early event in human hepatocarcinogenesis [31]. Cyclin D1 (Ccnd1) and cyclin-dependent kinase inhibitor 1C (Cdkn1c = p57) are critical for the cell cycle, including G1 progression and G1/S transition. Cyclin D1 has been shown to be amplified in 10 to 20% of HCCs.

### Implication of CGMA analysis

Chromosomal aberrations are common in cancers, particularly in advanced stages. CGH has been employed to determine gross DNA gains and losses at chromosomal and sub-chromosomal levels [10]. CGH, however, is time-consuming, and lacks the resolution and sensitivity to detect changes at the gene level; for example, CGH is unable to detect copy number changes within narrow regions of chromosomes (alternation of <1 Mb). It fails to identify putative tumor-suppressor genes or oncogenes [32]. These limitations might be overcome by using CGMA [12]. CGMA identifies cytogenetic regions containing unidirectional gene expression biases. Such region-dependent expression change may be the result of allelic imbalances commonly found in liver and other cancers. Evidence shows that DNA copy number alterations (deletion, low, mid and higher-level amplification) with an average 2-fold change in DNA copy number corresponds about a 1.5-fold change in mRNA level [33]. Therefore, CGMA based on microarray data measuring mRNA level could be related to DNA level.

Using CGMA, we identified 15 out of 46 (23 p and 23 q arms) human chromosomal regions that could be involved in liver cancer development, maintenance and progression. These chromosomal aberrations are consistent with the CGAP report for 10 out of 15 chromosome regions by karyotypes (Table 2). Although CGAP database (Table 2) cites no evidence of involvement of chromosome 19 in human liver cancer, we found that genes in both chromosome 19q and 19p are significantly down-regulated for three out of four tumor samples. In addition, there is also a block of genes in 19q that corresponds to rat 1q (Table 1) while a large number of differentially expressed genes also occurs in 19p using the Chromosome Plot (Figure is not shown). Analysis of both human 19p and q suggests the possible relevance of the chromosome in human liver cancer. The genes significantly altered in rat micorarray corresponding to human 19p13 are JunB, Rab8a (Mel), Tnfsf9 and Dnmt1. Further investigation is required to confirm their relevance to human HCC. Comparing with the findings by Crawley et al [12], we predicted chromosomal gains for five (12q, 16, 17q, 19p and 20q) out of eight of those reported by Crawley who carried out CGMA with human HCC gene expression arrays. Both our analysis and that of Crawley's suggest the importance of 19p in the human liver cancer, a region of aberration not previously discovered with CGH analysis. These results indicate human 19p as a region of aberration not previously discovered with CGH analysis.

### Implications of pathway analysis

Pathways are the best vehicle for interpretation of biological functions of genes. An important goal of modern biology is to identify the interaction and regulatory networks among biological molecules. A logical approach is to analyze the gene expression changes in the context of known biological pathways [34,35]. A number of human pathways were found to be significantly altered using the Fisher's Exact Test by comparing the number of genes with altered expression in a pathway to the number of genes on the chip in the same pathway.

We inferred which pathways are involved in human liver cancer from the differentially expressed genes in the transgenic rat liver cancer model. It is important to point out that the statistically significant pathways identified in this process were solely based on the analysis of microarray data together with the Orthologene and Pathway Libraries in ArrayTrack, and thus required no *a priori *knowledge regarding cancer genes and the pathways that they control. The results of the pathway analysis given in Table 3 include those involved in apoptosis, cell cycle, cell growth and differentiation and others that are significant in liver cancer.

Most of the altered pathways are involved in cell cycle regulation. In cancer, disruption of cell cycle regulation is accomplished by coordinating the activity of cyclin-dependent kinases, checkpoint controls, and DNA repair pathways, which, when perturbed lead to uncontrolled cell growth [27]. Not surprisingly, our studies support that P53 and Rb signaling pathways as well as cyclin mediated pathway including G1-S checkpoints are altered. Both p53 and Rb are tumor-suppressor genes, and their products are transcription factors that respond to a variety of stress signals and are often associated with the progression of neoplastic diseases. The transgenic model implies that both P53 and Rb signaling pathways could be disrupted in the human liver cancer. Without P53 and Rb, cell cycle arrest and/or programmed cell death (apoptosis) are inhibited, leading to accumulation of mutations and genetic instability. Since P53 is deactivated in this transgenic model, we also observed that the pathway that influences Ras and Rho proteins during G1 to S transition is altered. Ras is a proto-oncogene that is involved in multiple signal transduction pathways transmitting pro-proliferative signals to the nucleus, while Rho proteins are members of the extended Ras family that modulate gene expression, cell cycle progression, and cell proliferation and survival.

Categoies of altered pathways are associated with growth and differentiation. Genes in the ECM (extracellular matrix) and Integrin mediated signaling pathway have been reported to be over-expressed in human HCC, though the mechanism is not fully understood [36]. In addition, an excess of TGF-beta is thought to overwhelm the cell in two ways. First, it promotes the overgrowth of blood vessels. Second, excess TGF-beta suppresses T cells and other components of the immune system that would normally attack aberrant cells.

The human-relevant liver cancer pathways based on SV 40 transgenic rat liver model are confirmed by reports on human liver cancer models. Therefore, the pathway analysis using Fisher's Exact Test is novel and efficient.

## Conclusion

We presented several bioinformatics approaches to extrapolate microarray data involving rat liver cancer to the human. Microarray has been widely used in many fields of medical and biological research. The current challenge of bioinformatics of microarray is no longer to identify a list of differentially expressed genes, but to develop effective bioinformatics processes and tools for data interpretation and knowledge discovery. In this study, we first developed a Chromosome Plot that provides a compact visual summary of gene expression data at the level of chromosomal location for identification of altered chromosomal regions. This tool facilitates cross-species comparison. The information available in ArrayTrack on gene ontology, gene orthologs and gene pathways was then used to interpret the microarray data. Finally, the CGMA bioinformatics tool was uesd to infer HCC chromosomal aberrations in the human based on microarray data from rat. The important lesson of this study is how to limit the information using bioinformatics resources and statistical means to present an unbiased (or statistical) view to interpret microarray results with respect to genes, pathways, chromosomes and functions. Based on a thorough bioinformatics analysis, we found that the albumin-SV40 transgenic rat is a useful animal model for prediction of human liver cancer.

## Authors' contributions

HF did all calculations and analysis using bioinformatics tools, and wrote the first draft manuscript. WT guided the analysis and helped draft the manuscript. YD had the original idea for the liver cancer and liver toxicity study, provided original tissue, and designed the microarray analysis experiment; provided insightful information concerning liver cancer. HCP provided discussion and the transgenic rats. SHY provided the microarray data. JMY coordinated the collaboration and microarray experiment. WT, HH, QX, LS, XC and RP were involved in the discussion of the data analysis. RP and LS assisted with writing the manuscript. All authors read and approved the final manuscript.

## Figures and Tables

**Table 1:** Seven blocks of the significant genes from the rat 1q conservated across rat, mouse and human. The log2-transformed expression of average fold change (average over four tumor samples) for each gene is given in column two with direction up or down indicated by the sign, where genes with average fold change greater than an arbitrary +1.87 and less than -1.87 are highlighted.

**Genes**	**Average Fold changes Log2**	**Cytogenetic location (cM)**			**Descriptions**
			
		**Rat**	**Mouse**	**Human**	
Tctex1	**2.05**	1q11	17 3.1		t-complex testis expressed 1
Slc22a1	-0.32	1q11-q12	17 7.34	6q26	solute carrier family 22, member 1
Igf2r	**2.53**	1q11	17 7.35	6q26	insulin-like growth factor 2 receptor
Psmb1	1.35	1q12	17 8.25	6q27	proteasome (prosome, macropain) subunit, beta type 1

Calm3	**3.37**	1q22	7 4.0	19q13.2-q13.3	calmodulin 3 carcinoembryonic antigen-related cell adhesion
Ceacam1	-0.40	1q21-q22	7 5.5	19q13.2	molecule 1
Grik5	**1.92**	1q21	7 6.5	19q13.2	glutamate receptor, ionotropic, kainate 5
Zfp36	1.04	1q21	7 10.2	19q13.1	zinc finger protein 36
Usf2	0.98	1q21	7 11.0	19q13	transcription factor USF2
Sth2	-1.60	1q21.3-q22.1	7 A1		sulfotransferase, hydroxysteroid preferring 2
Rps16	**2.55**	1q21	7 A3	19q13.1	ribosomal protein S16
Rps19	**2.64**	1q21	7 A3	19q13.2	ribosomal protein S19
Pafah1b3	1.62	1q21	7 B1	19q13.1	platelet-activating factor acetylhydrolase, isoform 1b, alpha1 subunit
Ech1	0.92	1q21	7 B	19q13.1	enoyl coenzyme A hydratase 1
Atf5	-0.75	1q22	7 B3	19q13.3	activating transcription factor 5
Myd116	-0.86	1q22	7 B3		myeloid differentiation primary response gene 116
Nkg7	-0.88	1q22	7 B3	19q13.41	natural killer cell group 7 sequence
Nucb	0.96	1q22	7 B3		nucleobindin
Pold1	**2.06**	1q22	7 23.0	19q13.3	DNA polymerase delta, catalytic subunit
Emp3	0.95	1q22	7 24.5	19q13.3	epithelial membrane protein 3
Tead2	**2.18**	1q22	7 23.0	19q13.3	TEA domain family member 2
Bax	1.65	1q31.2	7 23.0	19q13.3-q13.4	Bcl2-associated X protein

Fzd4	0.55	1q32	7 44.5	11q14.2	frizzled homolog 4 (Drosophila)
Nox4	0.85	1q32	7 D3	11q14.2-q21	NADPH oxidase 4
Rps3	-2.43	1q32	7 E1	11q13.3-q13.5	ribosomal protein S3
Skd3	1.28	1q32	7 E1	11q13.3	suppressor of K+ transport defect 3
Thrsp	0.23	1q32	7 E1	11q13.5	thyroid hormone responsive protein

Ccnd1	-0.43	1q42	7 72.3	11q13	cyclin D1
Ppp1ca	**1.92**	1q43	7 E3-F2	11q13	protein phosphatase 1, catalytic subunit, alpha isoform
Cfl1	**2.83**	1q43	19 A	11q13	cofilin 1
Fen1	**4.79**	1q43	19 A	11q12	Flap structure-specific endonuclease 1
Gstp2	1.43	1q42	19 0.0		glutathione S-transferase, pi 2
Cpt1a	-0.42	1q43	19 2.0	11q13.1-q13.2	carnitine palmitoyltransferase 1, liver
Plcb3	1.39	1q43	19 2.5	11q13	phospholipase C, beta 3
Chk	1.06	1q42	19 3.0	11q13.1	choline kinase
Emk(Mark2)	1.63	1q43	19 3.0	11q12-q13	MAP/microtubule affinity-regulating kinase 2 membrane-spanning 4-domains, subfamily A, member
Ms4a2	-0.83	1q43	19 8.0	11q13	2

Ilk	1.47	1q33	7 E1	11p15.5-p15.4	integrin-linked kinase
Arhg	1.66	1	7 E2	11p15.5-p15.4	Ras homolog gene family, member G
Mrpl17	0.94	1q33	7 E3	11p15.5-p15.4	mitochondrial ribosomal protein L17
Arntl	**3.58**	1q34	7 52.0	11p15	aryl hydrocarbon receptor nuclear translocator-like
Cd81	-0.60	1q41	7 69.3	11p15.5	CD 81 antigen
Tssc3	**2.00**	1q41	7 69.5	11p15.5	pleckstrin homology-like domain, family A, member 2
Tnni2	0.97	1q41	7 70.0	11p15.5	troponin 1, type 2
Cdkn1c(p57)	**4.28**	1q42	7 69.49	11p15.5	cyclin-dependent kinase inhibitor 1C, p57

Coro1a	1.10	1q36	7 62.5	16p12.1	coronin, actin binding protein 1A
Mapk3	1.80	1q36	7 61.0	16p12-p11.2	protein kinase, mitogen activated 3 (extracellular-signal-regulated kinase 1, ERK1)
Maz	**1.97**		7 F3	16p11.2	MYC-associated zinc finger protein (purine-binding transcription factor)
Ppp4c	1.23	1q36	7 F3	16p12-16p11	protein phosphatase 4 (formerly X), catalytic subunit
Umod	-0.88	1q36-q37	7 55.0	16p13.11	uromodulin
Fgfr2	-0.68	1q37	7 63.0	10q26	fibroblast growth factor receptor 2
Cyp2e1	-0.08	1q41-42	7 68	10q24.3	cytochrome P450, family 2, subfamily e, polypeptide 1
Echs1	-0.38	1q41	7 F4	10q26.2-q26.3	enoyl Coenzyme A hydratase, short chain 1
Oat	-0.12	1q41	7 63.0	10q26	ornithine aminotransferase

Scd1	0.40	1q54	19 43.0		stearoyl-Coenzyme A desaturase 1
Pitx3	0.95	1q54	19 46.5	10q25	paired-like homeodomain transcription factor 3
Add3	1.81	1q55	19 47.0	10q24.2-q24.3	adducin 3, gamma
Cspg6	**2.73**	1q55	19 D2	10q25	chondroitin sulfate proteoglycan 6
Cyp17	-0.64	1q55	19 46.0	10q24.3	cytochrome P450, subfamily 17
Pnliprp1	-0.87	1q55	19 29.0	10q26.12	pancreatic lipase related protein 1
Pdcd4	1.01	1q55	19 20.0	10q24	programmed cell death 4

**Table 2:** Summary of the Z statistics for human chromosomes for each test sample from the CGMA analysis. A Z-statistics of 1.96 corresponds to α = 0.05, or 95% confidence that the expression bias in the chromosome was not due to chance. A Z-statistic of 2.58 corresponds to 99% confidence. Chromosomes exhibiting unidirectional bias with at least 95% confidence have positive sign denoting up-regulation or negative sign for denoting down-regulation.

**Chromosome Region**	**Adenoma sample 1**	**Adenoma sample 2**	**Carcinoma sample 1**	**Carcinoma sample 2**	**Karyotype results in CGAP # Citations (# Patients)**
1.p	**2.06**	**2.67**	-1	1.5	1 (1)
7.p	0.82	**2.45**	-0.58	1.34	8 (34)
9.p	1.41	1.41	**-2.24**	**-2**	5 (23)
11.p	1	**2.53**	0.82	0.82	4 (10)
11.q	0.63	**2.29**	0.23	0	4 (10)
12.p	1.63	**2.24**	0	-1	2 (4)
12.q	**2.13**	**2.2**	**1.96**	1.94	2 (4)
14.-	1.73	**2.84**	**2.24**	**2.56**	
16.-	**2.84**	**3.13**	0	0	1 (1)
17.q	0.65	**2**	0.5	0	1 (6)
19.p	**2.83**	**3.15**	**2.84**	1.67	
19.q	**2.2**	**2.83**	**2.83**	1.7	
20.q	**3.27**	0.71	1.6	1.81	5 (20)
21.-	**2.53**	**3**	0	1.67	
22.-	1.94	**2.67**	0.71	1.63	

**Table 3:** Pathway analysis of 2223 significant genes using a Fisher's Exact Test identified the listed pathways (p < 0.05) that might be related to human liver cancer.

Pathways	The number of significant genes found in a pathway divided by the genes from the chip found in the same pathway	P value
**Apoptosis**		
TGF Beta Induced apoptosis	18/35	0.00055
**Cell cycle**		
P53 signaling pathway	11/21	0.0079
Regulation of P27 during cell cycle progression	6/8	0.0036
Influence of Ras and Rho Protein on G1 to S transition	9/12	0.00031
ECM and Integrin mediated signaling pathway	6/10	0.017
Rb signaling pathway	10/13	0.0001
Cyclin mediated Pathway	13/19	0.000062
G1-S checkpoint	15/21	0.0000072
**Growth and differentiation**		
Insulin signaling pathway	8/16	0.035
TGF beta signaling pathway	7/13	0.021
**Amino Acid/Metabolic pathway**		
Methionine Metabolism	4/5	0.014
**Other Signaling pathways**		
PKC-mediated pathway	8/13	0.0047
Heregulin signaling pathway	14/34	0.027
AR Mediated Signaling Pathway	9/20	0.038
Apigenin Signaling Pathway	6/9	0.0085
